# Evaluating the diagnostic performance of [^18^F]ALF-NOTA-FAPI-04 PET/CT in gastric cancer: a comparative study with [^18^F]FDG PET/CT

**DOI:** 10.1007/s00330-024-11219-z

**Published:** 2024-11-28

**Authors:** Jinghui Lv, Kai Zheng, Chengzhi Jiang, Jian Yang, Xiang Peng, Hui Ye, Yanyin Zhang

**Affiliations:** 1 Department of Nuclear Medicine, Hunan Cancer Hospital/The Affiliated Cancer Hospital of Xiangya School of Medicine, Central South University, Changsha, China; 2 Department of PET-CT Center, Hunan Cancer Hospital/The Affiliated Cancer Hospital of Xiangya School of Medicine, Central South University, Changsha, China

**Keywords:** [^18^F]ALF-NOTA-FAPI-04, [^18^F]FDG, Gastric cancer, Fibroblast activation protein inhibitor, PET/CT

## Abstract

**Purpose:**

To compare the diagnostic value of [^18^F]ALF-NOTA-FAPI-04 positron emission tomography/computed tomography (PET/CT) and ^18^F-fluorodeoxyglucose (FDG) PET/CT in gastric cancer.

**Methods:**

This single-center retrospective analysis included 65 patients with gastric cancer who received both [^18^F]FDG and [^18^F]ALF-NOTA-FAPI-04 PET/CT for initial staging or restaging. Histopathological manifestations, typical imaging manifestations, follow-up imaging, and comprehensive clinical assessment were used as reference criteria. The uptakes of [^18^F]FDG and [^18^F]ALF-NOTA-FAPI-04 PET were compared using the Wilcoxon signed-rank test. McNemar’s test was employed to compare the diagnostic performance of the two imaging techniques.

**Results:**

A total of 65 patients were included (26 male and 39 female; mean age, 54.03 ± 10.41 years), Among them, 10 were newly diagnosed, 46 underwent radical gastrectomy, and 9 received only chemotherapy prior to the study. Compared with [^18^F]FDG PET/CT, [^18^F]ALF-NOTA-FAPI-04 PET/CT showed higher sensitivity in primary or recurrent tumors (100% vs. 64.52%, *p* < 0.001)), lymph node metastases (88.89% vs. 38.89%, *p* = 0.006), distant metastases (91.18% vs. 50%, *p* < 0.001). From the semi-quantitative evaluation, the Maximum standardized uptake value (SUVmax) and target-to-background ratio of [^18^F]ALF-NOTA-FAPI-04 PET/CT were significantly higher than that of [^18^F]FDG PET/CT in primary or recurrent tumors, lymph node metastases, and distant metastases (all *p* < 0.001).

**Conclusion:**

Our study results indicate that [^18^F]ALF-NOTA-FAPI-04 PET/CT outperforms [^18^F]FDG PET/CT in the detection of primary or recurrent tumors, lymph node metastasis, and distant metastasis in gastric cancer.

**Key Points:**

***Question*** *Early diagnosis and precise staging of gastric cancer are crucial for patient prognosis; however, current imaging techniques still face significant limitations.*

***Findings*** [^18^F]ALF-NOTA-FAPI-04 PET/CT demonstrated significantly higher sensitivity than [^18^F]FDG PET/CT in detecting primary or recurrent tumors and metastases in patients with gastric cancer.

***Clinical relevance*** [^18^F]ALF-NOTA-FAPI-04 PET/CT is an advanced imaging diagnostic technique that significantly enhances the diagnostic accuracy for gastric cancer and its metastatic lesions. This technology provides robust support for clinical decision-making, thereby improving the management of patients with gastric cancer.

## Introduction

Gastric cancer is the fifth most common cancer worldwide and ranks fourth in cancer-related deaths [1, 2]. Due to the inconspicuous symptoms of early gastric cancer, most patients are diagnosed at an advanced stage, resulting in poor prognosis [3]. Accurate diagnosis and staging play a crucial role in guiding the treatment of gastric cancer and significantly impact long-term prognosis. Multiple imaging modalities, including gastroscopy, endoscopic ultrasound, CT, and MRI, are employed in the diagnosis of gastric cancer. Nevertheless, these diagnostic approaches exhibit inherent false-negative results, affecting the accurate detection of lesions.

Fluorine 18 [^18^F] fluorodeoxyglucose positron emission tomography/computed tomography ([^18^F]FDG PET/CT) plays a pivotal role as a functional imaging modality, with significant implications in staging, restaging, treatment planning, and therapy response evaluation for various cancers [4]. Despite its utility, [^18^F]FDG PET/CT exhibits limitations in gastric cancer. Notably, primary lesions and metastatic foci in some types of gastric cancer, including mucinous, signet ring cell, and poorly differentiated adenocarcinoma, demonstrate lower [^18^F]FDG uptake [5–7]. Furthermore, the physiological uptake in the gastrointestinal tract and false-positive findings related to inflammation can complicate the identification of gastric cancer lesions.

Fibroblast activation protein (FAP), highly expressed in cancer-associated fibroblasts and minimally expressed in healthy tissues, represents a compelling and promising target for tumor imaging [8, 9]. Fibroblast activating protein inhibitor (FAPI) PET/CT demonstrates significant promise in gastrointestinal tumor imaging. Several studies have shown that [^68^Ga]Ga-FAPI PET/CT has a higher lesion detection rate and semi-quantitative parameters in gastrointestinal tumors compared to [^18^F]FDG PET/CT [10, 11]. Despite its potential, the use of [^68^Ga]Ga-FAPI is constrained by its short half-life (68 min), suboptimal spatial resolution, and insufficient availability of radionuclides from the ^68^Ge/^68^Ga generator. Conversely, [^18^F]ALF-NOTA-FAPI-04, a radiotracer labeled with [^18^F], can be mass-produced via a cyclotron and transported over long distances and has demonstrated excellent tumor imaging capabilities in various clinical evaluations [12, 13].

The aim of this study is to investigate the diagnostic value of [^18^F]ALF-NOTA-FAPI-04 PET/CT in gastric cancer and compare it with [^18^F]FDG PET/CT.

## Materials and methods

### Patients

This is a retrospective study that has been reviewed and approved by the institutional review board of the Hunan Cancer Hospital (2021 New Medical Technology Expedited Review (No. 02)) and registered at ClinicalTrials.gov (NCT06543108). Between December 2020 and August 2022, a total of 65 gastric cancer patients who underwent both [^18^F]ALF-NOTA-FAPI-04 and [^18^F]FDG PET/CT for staging and restaging were included in the study. All patients signed informed consent forms. Inclusion criteria were as follows: a. Gastric cancer confirmed through the histopathologic results (obtained from biopsy or surgery). b. Patients who underwent both [^18^F]ALF-NOTA-FAPI-04 and [^18^F]FDG PET/CT for initial staging and restaging within a two-week period, with no treatment administered between the two examinations. Patients who had more than two primary tumors, were aged less than 18 years, or were pregnant were excluded from the study. Primary tumors were diagnosed based on histopathological results obtained from biopsy or surgery. The diagnosis of lymph node metastasis and distant metastasis was based on histopathological manifestations, typical imaging manifestations, follow-up imaging, and comprehensive clinical assessment. Lesions determined as positive or negative by imaging standards were referenced from previous reports [14]. Because the participants had an advanced stage of the disease, only a few biopsies were performed for the suspected metastatic lesions.

### Preparation of [^18^F]FDG and [^18^F]ALF-NOTA-FAPI-04

The [^18^F] radionuclide was synthesized in situ by subjecting O-18-H2O to a 9.8 MeV proton bombardment using a GE MINItrace cyclotron (GE HealthCare). The FAPI-04 precursor was procured from PET Science and Technology CO., LTD. [^18^F]ALF-NOTA-FAPI-04 was labeled using the procedure detailed by Jiang et al [15]. The manufacturing of [^18^F]FDG followed the standard procedure, utilizing the coincidence [^18^F]FDG synthesis module (AIO, TRSIS). Both [^18^F]ALF-NOTA-FAPI-04 and [^18^F]FDG exhibited a radiochemical purity exceeding 95%. All radiotracers were sterile and pyrogen-free to meet the set criteria for human administration.

### PET/CT imaging

Patients were advised to fast for at least 6 h prior to the [^18^F]FDG PET/CT scan, and their blood glucose levels were below 11.1 mmol/L at the time of the [^18^F]FDG injection. Patients were instructed to pre-hydrate with 500 mL of water to facilitate the renal calyceal excretion and subsequent urinary elimination of [^18^F]FDG and [^18^F]ALF-NOTA-FAPI-04. The intravenously injected dose was calculated based on the patient’s weight (3.7 MBq (0.1 mCi)/kg for [^18^F]FDG/[^18^F]ALF-NOTA-FAPI-04). Data acquisition was performed using a hybrid PET/CT scanner (Discovery MI, GE HealthCare, Milwaukee, WI, USA) 1 h after intravenous administration. The protocol of [^18^F]FDG and [^18^F]ALF-NOTA-FAPI-04 PET/CT was the same as follows: A free-breathing CT scan was performed from the vertex of the skull to the mid thighs, and used for attenuation correction purposes as well as for anatomic location. The CT images were obtained using 64-slice helical CT with the following settings: 110 kV, 200–360mAs with automated dose modulation, a pitch of 0.984: 1, a noise index of 30, a gantry rotation of 0.5 s, and a slice thickness of 3.75 mm. Immediately after the CT scan, PET images were acquired covering the identical anatomical region. The PET acquisition time was set to 2 min per bed position, with 5–6 bed positions per patient (depending on patient size). List-mode PET data were reconstructed with a 256 × 256-pixel matrix, and the slice thickness was set to 3.75 mm. All the acquired data were transferred to the Advantage Workstation (AW 4.7, GE HealthCare), and were reconstructed using a Bayesian penalized likelihood reconstruction method (Q.Clear, GE HealthCare) with *β* = 750.

### Imaging analysis

All [^18^F]ALF-NOTA-FAPI-04 and [^18^F]FDG PET/CT examinations were independently reviewed by two certified nuclear medicine physicians with over 5 years of experience in nuclear oncology. Should there be any differences in diagnosis, a consensus was reached through discussion. Positive lesions were identified as areas of increasing radioactivity compared to the background uptake (excluding physiological tracer uptake and definitive benign diseases). Semi-quantitative analysis was performed on both [^18^F]ALF-NOTA-FAPI-04 and [^18^F]FDG PET/CT using Advantage Workstation. For primary and recurrent tumors, regions of interest (ROIs) were positioned on axial slices around avid-lesion regions and were automatically integrated into a 3D volume of interest (VOI). For metastatic lesions, VOIs were directly placed on the lesions. The maximum standardized uptake value (SUVmax) of the lesions was automatically calculated. A VOI was manually placed on the healthy tissue, and the mean standardized uptake value (SUVmean) was automatically measured. The target-to-background ratio (TBR) was calculated as the SUVmax of the lesion divided by the SUVmean of the aortic arch.

### Statistical analysis

Statistical analyses were conducted using SPSS Statistics (version 27.0; IBM) and R software (R 4.3.0). A two-tailed *p*-value of < 0.05 was considered to indicate statistical significance. Categorical variables are described as frequencies, and continuous variables are presented as mean ± SD or median (interquartile range (IQR)), depending on the distribution of values. The comparisons of sensitivity, specificity, negative predictive value (NPV), positive predictive value (PPV), and accuracy were performed using McNemar’s test. The differences in SUVmax and TBR between [^18^F]ALF-NOTA-FAPI-04 and [^18^F]FDG PET/CT were evaluated using the Wilcoxon signed-rank test (skewed variables).

## Result

### Patients characteristics

**Fig. 1:**
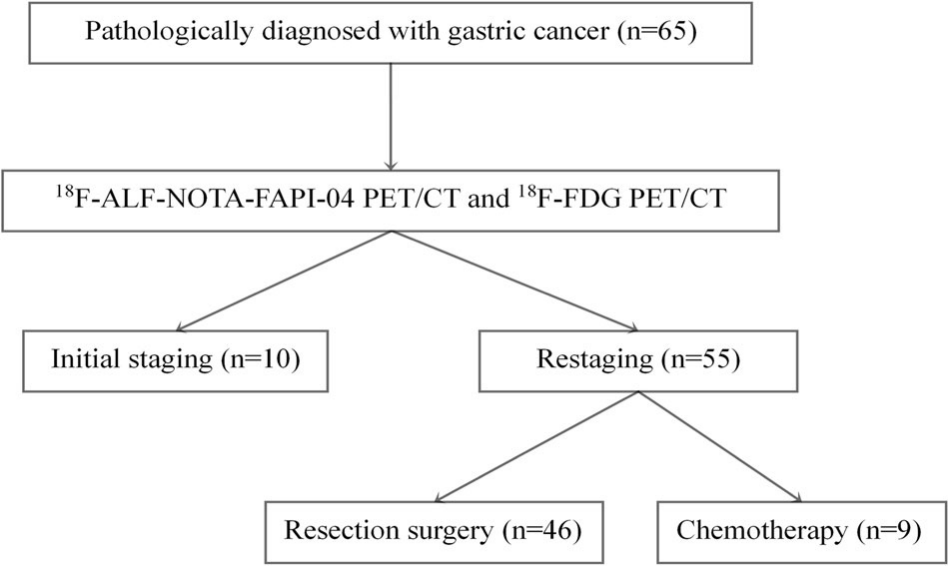
Study flowchart

**Fig. 2:**
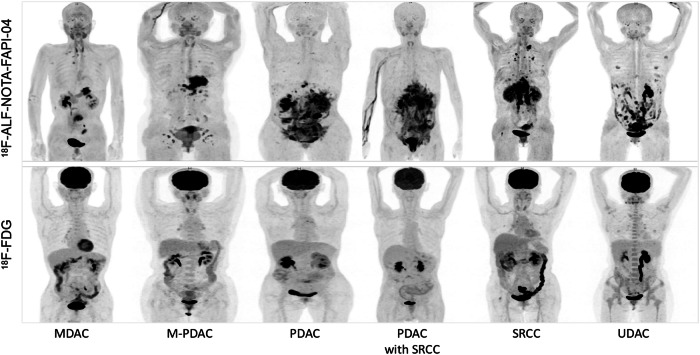
Representative MIP images of gastric cancer with various pathological types. [^18^F]ALF-NOTA-FAPI-04 PET images were superior to [^18^F]FDG PET images in terms of primary tumor and metastasis. MIP, maximum-intensity projection; MDAC, moderately differentiated adenocarcinoma; M-PDAC, moderately and poorly differentiated adenocarcinoma; PDAC, poorly differentiated adenocarcinoma; SRCC, signet ring cell carcinoma; UDAC undifferentiated adenocarcinoma

A total of 65 patients with gastric cancer (39 female; mean age, 54.03 ± 10.41 years) were enrolled in this study. There were 10 patients newly diagnosed with gastric cancer and 55 patients undergoing restaging post-treatment. The characteristics of the patients, along with the pathological types of gastric cancer, are detailed in Table 1. Figure 1 presents the study design as a flow diagram. Representative MIP images of gastric cancer with various pathological types are shown in Fig. 2.

**Table 1:** Patient characteristics

*N* = 65	Value
Age (years)^a^	54.03 ± 10.41 (29–72)
Sex	
Male	26
Female	39
Indication for PET	
Staging	10
Restaging	55
Patient status	
Treatment-naive	10
Resection surgery	46
Chemotherapy	9
Pathologic types	
Moderately ADC	5
Moderately and poorly ADC	10
Poorly ADC	23
Poorly ADC with SRCC	23
SRCC	3
Undifferentiated adenocarcinoma	1

^a^ Age is expressed as the mean ± SD, with range in parentheses. ADC adenocarcinoma, SRCC signet ring cell carcinoma

### Diagnostic performance of [^18^F]ALF-NOTA-FAPI-04 and [^18^F]FDG PET/CT for primary or recurrent tumors in gastric cancer

**Fig. 3:**
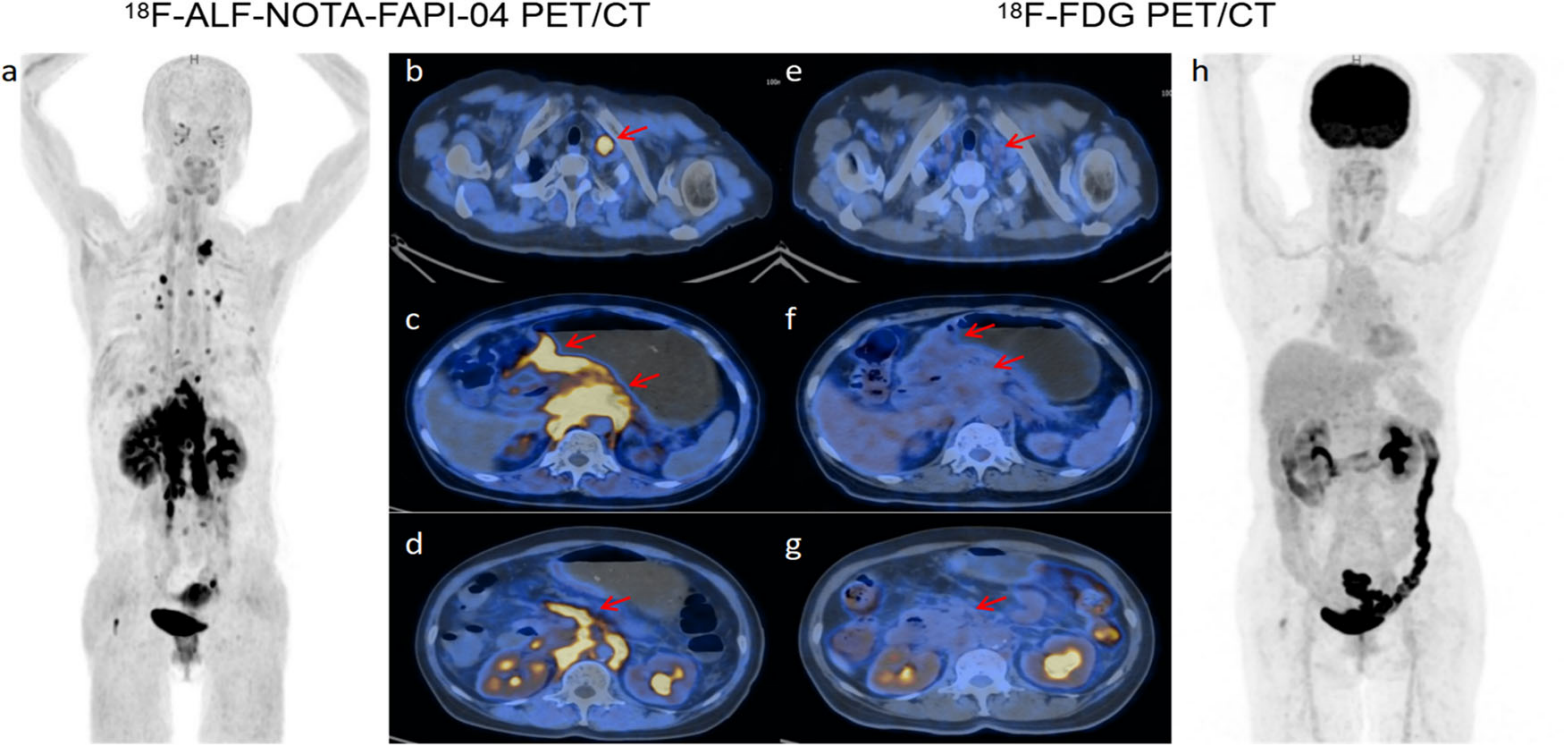
A 64-year-old female patient with signet ring cell carcinoma. The uptake of lesions in [^18^F]ALF-NOTA-FAPI-04 PET/CT (**a**–**d**) was higher than that in [^18^F]FDG PET/CT (**e**–**h**) (primary tumor SUVmax = 12.8 vs. 2.5; lymph node metastasis SUVmax = 25.0 vs. 1.8; peritoneal metastasis SUVmax = 26.5 vs. 3.1, respectively

**Fig. 4:**
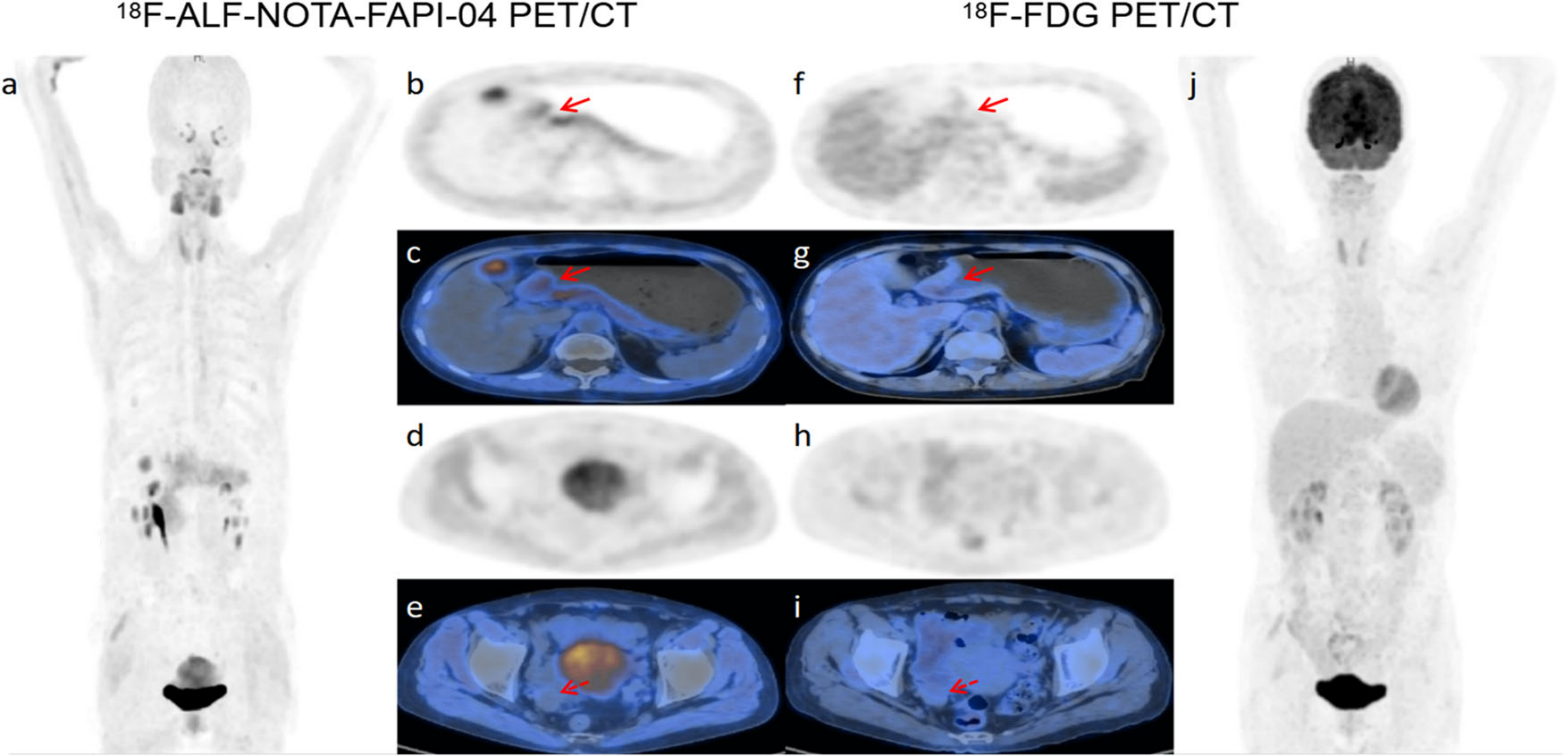
A 56-year-old female with poorly differentiated adenocarcinoma underwent follow-up examination after 10 cycles of chemotherapy. [^18^F]ALF-NOTA-FAPI-04 PET/CT (**a**–**c**) demonstrated moderate uptake in the gastric antrum and angulus (solid arrows, SUVmax = 3.9), while [^18^F]FDG PET/CT (**f**, **g**) showed mild uptake (solid arrows, SUVmax = 2.5). Postoperative pathology confirmed residual diffuse and multifocal moderate-poorly differentiated adenocarcinoma tissue. Surgical pathology of the right ovary also confirmed the presence of poorly differentiated adenocarcinoma, consistent with the Krukenberg tumor. Both [^18^F]ALF-NOTA-FAPI-04 PET/CT (**d**, **e**) and [^18^F]FDG PET/CT (**h**, **i**) revealed negative uptake (dotted arrows)

**Fig. 5:**
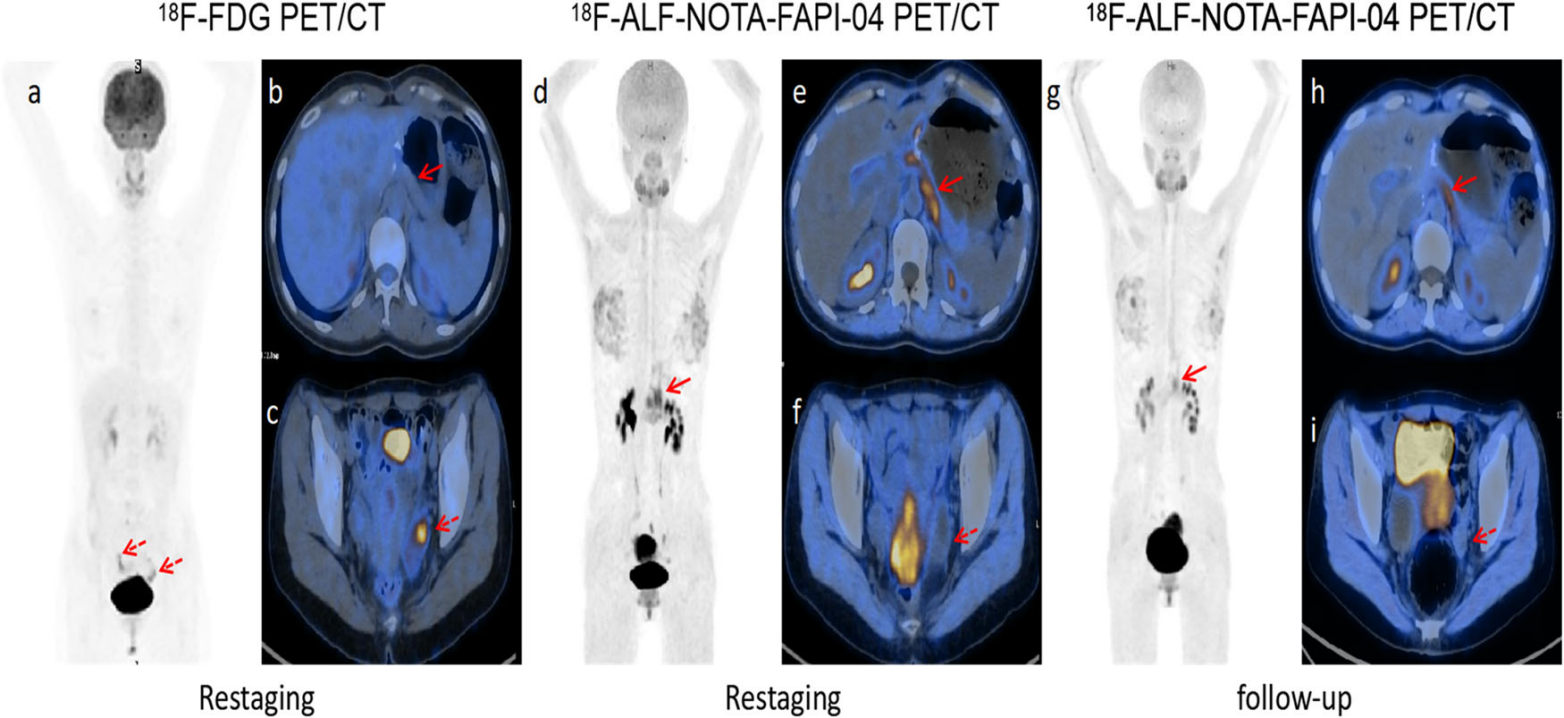
A 34-year-old female with a history of postoperative chemotherapy underwent PET/CT for restaging. Gastroscopy confirmed the recurrence of poorly differentiated adenocarcinoma containing SRCC in the gastric remnant. Restaging [^18^F]ALF-NOTA-FAPI-04 PET/CT (**d**, **e**) showed high uptake at the anastomotic site and residual gastric wall (solid arrows, SUVmax = 6.7), whereas [^18^F]FDG PET/CT (**a**, **b**) showed negative uptake (solid arrows, SUVmax = 1.2). The follow-up [^18^F]ALF-NOTA-FAPI-04 PET/CT (**g**, **h**) after 4 cycles of chemotherapy shows partial response(solid arrows, SUVmax = 3.9). [^18^F]FDG PET/CT (**a**, **c**) showed increased uptake in the ovaries bilaterally (dashed arrows, SUVmax = 10.8), but [^18^F]ALF-NOTA-FAPI-04 PET/CT (**d**, **f**, **g**, **i**) showed negative uptake (dashed arrows). After two years of follow-up CT, the lesion was confirmed to be physiologic uptake in the ovary

Based on pathological or follow-up findings, a total of 31 participants were diagnosed with primary or recurrent tumors. Of these, 22 cases were confirmed through biopsy and surgical pathology. The diagnostic performances of [^18^F]ALF-NOTA-FAPI-04 and [^18^F]FDG PET/CT are summarized in Table 2. The [^18^F]ALF-NOTA-FAPI-04 PET/CT exhibits superior sensitivity (100% vs. 64.52%, *p* < 0.001), NPV (100% vs. 74.42%, *p* = 0.006), and accuracy (96.92% vs. 80%, *p* = 0.006) compared to [^18^F]FDG PET/CT in the diagnosis of primary or recurrent gastric cancer (Figs. 3–5). The SUVmax and TBR of the primary and recurrent lesions in gastric cancer were significantly higher in the [^18^F]ALF-NOTA-FAPI-04 PET/CT than in the [^18^F]FDG PET/CT (all *p* < 0.001) (Table 3).

**Table 2:** Diagnostic performance of [^18^F]FDG and [^18^F]ALF-NOTA-FAPI-04 PET/CT in gastric cancer

	True positive (N)	True negative (N)	False positive (N)	False negative (N)	Sensitivity (%)	Specificity (%)	Accuracy (%)	PPV (%)	NPV (%)
Primary and recurrent tumors									
[^18^F]FDG	20	32	2	11	64.52	94.12	80	90.91	74.42
[^18^F]ALF-NOTA-FAPI-04	31	32	2	0	100	94.12	96.92	93.94	100
*p-*value					< 0.001	1.000	0.006	1.000	0.006
Lymph node metastases									
[^18^F]FDG	7	43	4	11	38.89	91.49	76.92	63.64	79.63
[^18^F]ALF-NOTA-FAPI-04	16	47	0	2	88.89	100	96.92	100	95.92
*p-*value					0.006	0.125	0.002	0.039	0.029
Distant metastases									
[^18^F]FDG	17	29	2	17	50	93.55	70.77	89.47	63.04
[^18^F]ALF-NOTA-FAPI-04	31	30	1	3	91.18	96.77	93.85	96.88	90.91
*p-*value					< 0.001	1.000	0.001	0.638	0.011

*N* number of patients, *PPV* positive predictive value, *NPV* negative predictive value

McNemar’s test was used to compare whether there were significant differences in sensitivity, specificity, accuracy, PPV, and NPV between the two groups

**Table 3:** Comparative results of semi-quantitative parameters between [^18^F]FDG and [^18^F]ALF-NOTA-FAPI-04 PET/CT in gastric cancer

Tumor lesions and parameters	[^18^F]FDG	[^18^F]ALF-NOTA-FAPI-04	*p-*value
Primary and recurrent tumors			
No. of lesions	20	31	
SUVmax	2.94 (2.12–4.41)	11.27 (8.90–14.72)	< 0.001
TBR	2.25 (1.59–4.37)	13.36 (7.48–18.33)	< 0.001
Lymph node metastases			
No. of lesions	32	129	
SUVmax	2.03 (1.23–5.14)	10.27 (8.51–20.76)	< 0.001
TBR	1.59 (0.97–3.26)	14.61 (9.76–24.08)	< 0.001
Distant metastases			
in the totality			
No. of lesions	30	63	
SUVmax	2.14 (1.57–4.22)	12.32 (9.54–17.02)	< 0.001
TBR	1.88 (1.27–3.67)	14.45 (8.78–18.70)	< 0.001
Peritoneum^a^			
No. of lesions	14	28	
SUVmax	1.82 (1.51–3.61)	12.52 (9.61–15.54)	< 0.001
TBR	1.67 (1.19-2.58)	14.27 (9.44-18.06)	< 0.001
Ovary			
No. of lesions	10	14	
SUVmax	3.47 (2.84–8.70)	10.82 (5.46–12.44)	0.017
TBR	2.52 (2.13–9.00)	12.64 (5.70–18.25)	0.012
Bone^b^			
No. of lesions	4	19	NA
Abdominal wall^b^			
No. of lesions	2	2	NA

The Wilcoxon signed-rank test was used to compare the SUVmax and TBR between the two groups

Data are median (IQR), *FAPI* fibroblast activation protein inhibitor, *FDG* fluorodeoxyglucose, *SUVmax* maximum standardized uptake value, *TBR* target-to-background ratio, *NA* not applicable

^a^ Number of patients

^b^ There were only two patients of both bone and abdominal wall metastases, which were not statistically analyzed

### Diagnostic performance of [^18^F]FDG and [^18^F]ALF-NOTA-FAPI-04 PET/CT in lymph node metastasis

A total of 18 participants were diagnosed with lymph node metastases based on pathological or follow-up findings, with histopathological confirmation in 5 cases. In patient-based lymph node analysis, the [^18^F]ALF-NOTA-FAPI-04 PET/CT demonstrates higher sensitivity (88.89% vs. 38.89%, *p* = 0.006), PPV (100% vs. 63.64%, *p* = 0.039), NPV (95.92% vs. 79.63%, *p* = 0.029), and accuracy (96.92% vs. 76.92%, *p* = 0.002) compared to the [^18^F]FDG PET/CT. In lesion-based analysis, the [^18^F]ALF-NOTA-FAPI-04 PET/CT detects a greater number of lymph node metastases (129 vs. 32) compared to the [^18^F]FDG PET/CT. The SUVmax and TBR of the lymph node metastases were significantly higher in the [^18^F]ALF-NOTA-FAPI-04 PET/CT than in the [^18^F]FDG PET/CT (all *p* < 0.001) (Table 3).

### Diagnostic performance of [^18^F]FDG and [^18^F]ALF-NOTA-FAPI-04 PET/CT distant metastasis

**Fig. 6:**
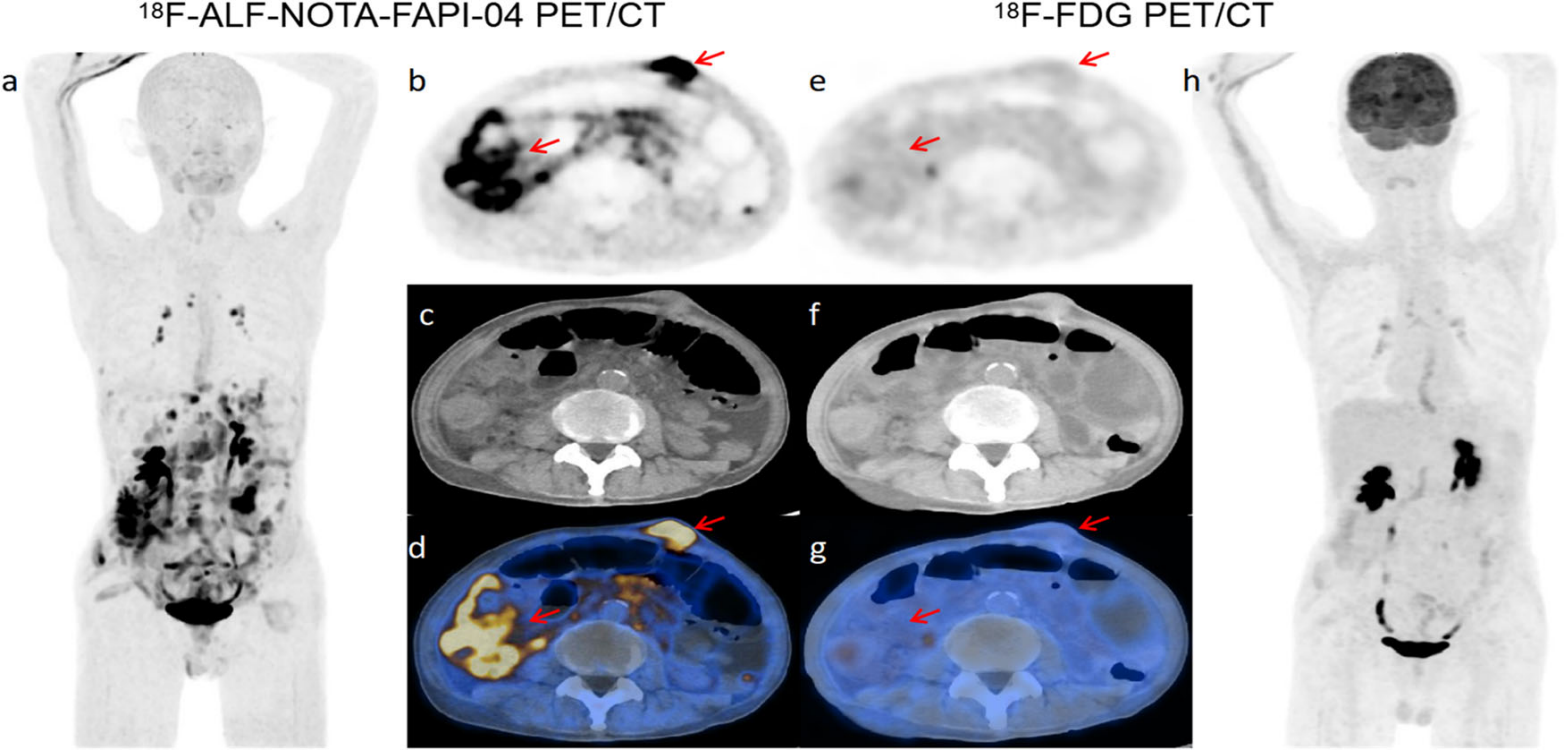
A 57-year-old male patient with poorly differentiated adenocarcinoma presented with abdominal distention and pain after postoperative chemotherapy. [^18^F]ALF-NOTA-FAPI-04 PET/CT (**a**–**d**) revealed diffuse high uptake in the peritoneum (SUVmax = 13.3) and the left anterior lower abdominal wall (SUVmax = 20.2), whereas [^18^F]FDG PET/CT (**e**–**h**) demonstrated mild uptake in the peritoneum (SUVmax = 2.1) and the left anterior abdominal wall (SUVmax = 2.2)

Based on a combination of pathology and imaging, 34 participants were identified with distant metastases, of which 9 were confirmed through biopsy and surgical pathology. In patient-based analysis, [^18^F]ALF-NOTA-FAPI-04 PET/CT demonstrated superior sensitivity (91.18% vs. 50%, *p* < 0.001), NPV (90.91% vs. 63.04%, *p* = 0.011), and accuracy (93.85% vs. 70.77%, *p* = 0.001) compared to [^18^F]FDG PET/CT in the diagnosis of distant metastases. For lesion-based analysis, the number of lesions in the peritoneum, ovaries, and bone detected by [^18^F]ALF-NOTA-FAPI-04 PET/CT was greater than that detected by [^18^F]FDG PET/CT (Table 3). The SUVmax and TBR of distant metastatic lesions were significantly higher in the [^18^F]ALF-NOTA-FAPI-04 PET/CT than in the [^18^F]FDG PET/CT (all *p* < 0.001)(Table 3) (Fig. 6).

## Discussion

In this study, we compared the diagnostic performance of [^18^F]ALF-NOTA-FAPI-04 PET/CT and [^18^F]FDG PET/CT in gastric cancer. Our findings revealed that [^18^F]ALF-NOTA-FAPI-04 PET/CT outperformed [^18^F]FDG PET/CT for detecting primary or recurrent tumors, lymph node metastases, and distant metastases. The SUVmax and TBR of lesions measured by [^18^F]ALF-NOTA-FAPI-04 PET/CT were significantly higher, enhancing visibility and reducing missed diagnoses.

FAPI, as a novel tumor imaging agent, demonstrates significant potential in the diagnosis and treatment of gastrointestinal tumors. Fu et al [6] analyzed 61 patients with primary gastric cancer and found that the detection rate of primary lesion by [^68^Ga]Ga-FAPI-04/^18^F-FAPI-42 PET/CT was significantly higher than that of [^18^F]FDG PET/CT(95.1% vs. 73.8%, *p* < 0.001). This result is consistent with our study. Additionally, our study found that [^18^F]ALF-NOTA-FAPI-04 PET/CT was more sensitive in diagnosing lymph node metastasis and distant metastasis in gastric cancer. Previous studies have also reported the excellent performance of FAPI PET/CT in detecting primary tumors and metastatic lesions in the gastrointestinal tract [16–18]. Therefore, [^18^F]ALF-NOTA-FAPI-04 PET/CT can provide more precise information in the evaluation of gastric cancer and assist in developing clinical treatment plans.

In our study, [^18^F]ALF-NOTA-FAPI-04 PET/CT identified two false-positive cases of anastomotic recurrence, which were later confirmed by biopsy pathology to be anastomotic inflammation. This may be associated with inflammation and the high expression of FAP in activated fibroblasts at the surgical site [19]. Additionally, one of these cases also showed high uptake on [^18^F]FDG PET/CT. Therefore, the differentiation between benign and malignant gastric cancer should not only consider the degree of radioactivity uptake but also incorporate other radiological features and clinical evidence. In lymph node analysis, [^18^F]ALF-NOTA-FAPI-04 PET/CT showed higher SUVmax and better detection than [^18^F]FDG PET/CT. However, [^18^F]ALF-NOTA-FAPI-04 PET/CT missed two cases of perigastric lymph node metastasis, which were later confirmed through postoperative pathological examination following gastric cancer surgery. Surprisingly, [^18^F]FDG PET/CT showed high uptake in the left supraclavicular lymph nodes in 4 patients, whereas [^18^F]ALF-NOTA-FAPI-04 PET/CT showed low uptake. Of these negative lymph nodes, 2 were confirmed by biopsy, and 2 were confirmed by follow-up CT. Previous case reports also suggest that [^68^Ga]Ga-FAPI may be superior to [^18^F]FDG in distinguishing reactive lymph nodes from metastatic lesions [20, 21]. However, Lan et al [22] found that [^68^Ga]Ga-DOTA-FAPI PET/CT showed consistent tracer uptake with [^18^F]FDG PET/CT in lymphadenitis. Therefore, for lymph node metastasis, the assessment by [^18^F]ALF-NOTA-FAPI-04 PET/CT still requires further validation with a larger sample size.

In this study, both [^18^F]ALF-NOTA-FAPI-04 PET/CT and [^18^F]FDG PET/CT diagnosed 2 patients with bone metastases, and follow-up CT showed progression of the lesions. However, [^18^F]ALF-NOTA-FAPI-04 PET/CT detected more bone metastases with a higher uptake (19 vs. 4), which is similar to the study by Chend et al [7]. Their study also demonstrated that for bone metastases, [^68^Ga]FAPI PET was less specific than [^18^F]FDG PET, as false-positive uptake of FAPI was more likely in cases of myelofibrosis and arthritis. In the diagnosis of ovarian metastases, 3 cases were confirmed by biopsy and surgical pathology. Among these, one patient with right ovarian metastasis showed negative uptake on both [^18^F]ALF-NOTA-FAPI-04 and [^18^F]FDG PET/CT (Fig. 4). Additionally, one patient exhibited bilateral ovarian lesions on [^18^F]FDG PET/CT but showed negative results on [^18^F]ALF-NOTA-FAPI-04 PET/CT. However, a two-year follow-up CT did not show any significant ovarian metastases in this case (Fig. 5). Qin et al [10] reported a false-positive case of bilateral ovarian uptake on both [^18^F]FDG PET/CT and [^68^Ga]FAPI PET/MR, which was surgically confirmed as corpus luteum. Therefore, FAPI PET still has significant limitations in detecting ovarian metastases. [^18^F]ALF-NOTA-FAPI-04 PET/CT demonstrated significant superiority over [^18^F]FDG PET/CT in detecting peritoneal metastases; this was confirmed by biopsy pathology in three patients, aligning with the results of a previous study [6, 23]. This may be attributed to tumor invasion into peritoneal tissue, which induces significant fibrosis in the peritoneal mesenchyme.

In our study, one patient with residual gastric recurrence who was FAPI-positive and FDG-negative underwent a second follow-up [^18^F]ALF-NOTA-FAPI-04 PET/CT scan after four cycles of chemotherapy, which showed partial remission (Fig. 5). The research of Kuten et al [24] monitored the chemotherapy response for peritoneal carcinomatosis using baseline and follow-up [^68^Ga]Ga-FAPI-04 PET/CT in two cases. These findings demonstrate the potential value of FAPI PET/CT in monitoring tumor response, but research in this area remains limited.

Due to the rapid clearance of [^18^F]ALF-NOTA-FAPI-04 by the kidneys, along with its lower physiological uptake in normal organs and higher TBR, FAP-targeted radiopharmaceutical therapy (FAP-RPT) emerges as an effective method to precisely deliver radiation to FAP- and stroma-rich tumor lesions while minimizing damage to surrounding normal tissues [25]. This approach offers a promising therapeutic target for a variety of malignant tumors. The strong expression of FAP in tumor lesions (SUVmax of at least 10) suggests their suitability for FAP-RPT treatment [26]. FAPI with ^177^Lu for the treatment of various solid tumors has been reported, demonstrating that FAP-RPT has good safety and efficacy [27, 28]. Additionally, Li et al [29] reported a case of a patient with recurrent bladder tumor and multiple metastases treated with [^177^Lu]Lu-FAPI-2286, which demonstrated significant remission on imaging and a marked reduction in symptoms after only one treatment cycle. The elevated SUVmax and TBR of [^18^F]ALF-NOTA-FAPI-04 observed in our study indicate that FAPI-targeted radioligand therapy holds promise as an innovative treatment for gastric cancer.

Our study also has several limitations. First, the limited sample size and selection bias may have an impact on the results. Additional studies with larger cohorts are required to verify the consistency of the results. The second shortcoming is that although the study included several common pathological types, [^18^F]ALF-NOTA-FAPI-04 PET/CT are incapable of fully depicting the uptake pattern of all gastric cancer. Lastly, due to technical and ethical constraints, it was impossible to biopsy all lymph nodes and distant metastases, leading to a lack of a definitive pathological diagnosis. Employing an alternative approach in our study, we confirmed the metastatic lesions through typical imaging manifestations, follow-up imaging, and comprehensive clinical assessment.

In conclusion, this retrospective study revealed that the diagnostic performance of [^18^F]ALF-NOTA-FAPI-04 PET/CT in gastric cancer surpassed those of [^18^F]FDG PET/CT. The [^18^F]ALF-NOTA-FAPI-04 PET/CT shows significantly higher radiotracer uptake and TBR than [^18^F]FDG PET/CT. [^18^F]ALF-NOTA-FAPI-04 PET/CT may present as a more promising approach for evaluating gastric cancer, with the potential to enhance patient management in the future.

## Abbreviations

FAP: Fibroblast activation protein
FAPI: Fibroblast activation protein inhibitor
FAP-RPT: FAP-targeted radiopharmaceutical therapy
FDG: Fluorodeoxyglucose
IQR: Interquartile range
NPV: Negative predictive value
PET: Positron emission tomography
PPV: Positive predictive value
SRCC: Signet ring cell carcinoma
SUVmax: Maximum standardized uptake value
SUVmean: Mean standardized uptake value
TBR: Target-to-background ratio
